# Parents' perspectives and behaviors regarding their child's access to alcohol: Variation by race/ethnicity, socioeconomic status, and neighborhood

**DOI:** 10.1111/acer.15498

**Published:** 2024-12-19

**Authors:** Carolyn E. Sartor, Shawn J. Latendresse, Kristina M. Jackson, Mai‐Ly N. Steers, Sharon Lipperman‐Kreda, Tim Slade, Tammy Chung

**Affiliations:** ^1^ Rutgers Institute for Health, Health Care Policy and Aging Research Brunswick New Jersey USA; ^2^ Rutgers Addiction Research Center Rutgers Robert Wood Johnson Medical School New Brunswick New Jersey USA; ^3^ Baylor University Waco Texas USA; ^4^ Duquesne University Pittsburgh Pennsylvania USA; ^5^ Prevention Research Center Pacific Institute for Research and Evaluation Berkeley California USA; ^6^ The Matilda Centre for Research in Mental Health and Substance Use Sydney University Sydney New South Wales Australia

**Keywords:** alcohol access, parental rules, race/ethnicity, socioeconomic status, youth

## Abstract

**Background:**

Setting rules about alcohol use and minimizing its availability in the home are known effective parent‐level strategies for reducing underage drinking risk. However, parents' restrictions and their perceptions of their child's alcohol access have rarely been considered in combination (e.g., determining if rule‐setting consistently accompanies perceived easy access), despite the potential to inform targeted prevention. The current study identified patterns in six parent‐reported indicators of their child's alcohol restrictions and access and characterized them with respect to race/ethnicity, socioeconomic status, community type (urban, suburban, or rural), and neighborhood (dis)advantage.

**Methods:**

Latent profile analysis was applied to Follow‐up Year 2 data from the parents of Black, Latinx, and White participants in the Adolescent Brain Cognitive Development Study (*n* = 9586; youth mean age = 12.05; 47.50% girl, 51.32% boy, 0.32% other gender; 14.29% Black, 25.97% Latinx, and 59.74% White) to derive distinct profiles.

**Results:**

Four profiles (subgroups) emerged: High Restrictions/No Drinkers in Household (32.18%), Low Restrictions/High Access (29.58%), High Restrictions/High Access (26.38%), and High Restrictions/Low Access (11.86%). Black and Latinx youth and parents with relatively low educational attainment and income were overrepresented in the High Restrictions/No Drinkers in Household and High Restrictions/Low Access subgroups. By contrast, the low restrictions subgroups were composed primarily of parents of White youth living in advantaged neighborhoods.

**Conclusions:**

Findings support the notion that parents' perspectives and behaviors around youth alcohol access cannot be divided simply into restrictive and permissive. Further, the observed differences by demographic and neighborhood factors suggest the value of tailoring parent‐level prevention approaches to consider community norms.

## Graphical abstract

Parental restrictions on and perceptions of their pre‐adolescent child's access to alcohol (e.g., rule setting, alcohol storage in the home) vary such that high restrictions may pair with either high or low access to alcohol, forming distinct profiles. Furthermore, certain profiles are more common in some racial/ethnic groups or types of communities. For example, Black youth are overrepresented in the profile characterized by no household drinkers and the high restrictions/high access profile is most commonly observed in suburban communities.

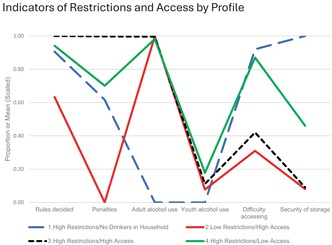

## INTRODUCTION

Restricting youth alcohol access is a well‐known protective parenting strategy against early alcohol use. Establishing strict rules about use in the household has consistently been linked to reduced likelihood of initiation during adolescence (Bo et al., 2018; Sharmin et al., 2017). Limiting the physical availability of alcohol, such as securely storing alcohol in the house, is also known to reduce risk for underage drinking (Harding et al., 2016). Despite the well‐established and face valid effectiveness of these strategies, which fall under the parenting framework “alcohol‐specific parenting” (Jacob & Johnson, 1997), they are not universally implemented (Janssen et al., 2014).

Parents' perceptions of their child's ease of access to alcohol likely impacts their thinking about the need for restrictions on alcohol use in the home. Although commonly queried of youth in substance use studies (including in the annual Monitoring the Future Study: Miech et al., 2023), youth's ease of access to alcohol is rarely asked of parents, with only a few notable exceptions (Cox et al., 2018; Ennett et al., 2016). As a consequence, relatively little is known about parents' beliefs about their child's access to alcohol. The perception that their child can easily acquire alcohol has the potential to result in posing high restrictions to reduce access, consistent with evidence that awareness of their child's drinking may lead to greater imposition of restrictions (van den Eijnden et al., 2011). The reverse might also be possible; parents believing that their child can easily obtain alcohol could lead to permissive attitudes and behaviors. Indeed, studies have shown that parents may adopt more permissive attitudes toward youth alcohol use when they learn of their child's drinking (Glatz et al., 2012) and subsequently are less likely to enforce strict rules about alcohol use (Koutakis et al., 2008).

A more nuanced understanding of the context in which parents' perceptions of their child's access to alcohol coincides with their rule‐setting is critical for delivering parent‐level prevention strategies to limit their child's access. An investigation by Ennett et al. (2016) addressed this broad goal. They identified subgroups of parents of adolescents with respect to patterns of general parenting and alcohol‐specific parenting behaviors, including perceptions of their child's access to alcohol, rule‐setting about alcohol use, and their own alcohol use. Latent profile analyses revealed four profiles, two characterized as “conservative” and two as “tolerant,” with the two tolerant profiles differentiated by level of parents' alcohol use (i.e., tolerant paired alternatively with high versus low parental use). Notably, they found that relative to White parents, Black parents were less likely to fall into either “tolerant” class.

Consideration of potential distinctions in prevention targets, for example, rule‐setting, by sociodemographic factors such as race/ethnicity, socioeconomic status (SES), community type (i.e., urban vs. suburban or rural), and neighborhood advantage is key for shaping alcohol prevention efforts to meet the needs of different subpopulations. Alcohol use is more prevalent among White than Black or Latinx adults (SAMHSA, 2022), in high versus lower socioeconomic status families (Miech et al., 2023), disadvantaged versus advantaged neighborhoods (Segrin et al., 2023; Trucco et al., 2014), and suburban compared to urban or rural communities (Miech et al., 2023). As such, the prevention needs of youth from different backgrounds and communities may also differ.

Access to alcohol is naturally lower in households where adults abstain from use and abstention has been found to be highest among those with relatively low educational attainment (Kerr et al., 2016) and among Black adults, followed by Latinx adults (SAMHSA, 2022). Greater access to alcohol among Latinx youth compared to youth from other racial/ethnic groups has also been observed (Lardier Jr. et al., 2018). Regarding parental establishment of rules about their child's alcohol use, few studies have examined race/ethnicity differences. The very limited research, conducted over two decades ago, suggested that Black parents are more likely to create and enforce rules than their White counterparts (Johnson & Johnson, 1999), potentially out of concern about particularly harsh punitive responses from law enforcement or school officials (Fellner, 2009), reflective of systemic racism. Although Ennett et al. (2016) did not specifically assess parental establishment of rules about alcohol use, their finding that Black parents were less likely than White parents to fall into the “tolerant” profiles is consistent with potentially greater rule setting by Black parents.

Recognizing common parenting practices and perspectives among parents with similar racial/ethnic or socioeconomic backgrounds or who live in similar neighborhoods can facilitate the development of shared norms for restrictions on youth access to alcohol. Gilligan et al. (2014) have argued for capitalizing on parents' connections to other parents with respect to establishing rules around adolescent alcohol use, such that parents create norms within neighborhoods or schools through exposure to the attitudes and behaviors of other parents in the community. Capturing these patterns in perceptions and behaviors and characterizing them with respect to broad easily identifiable sociodemographic factors can lay the groundwork for a norm‐based approach to encouraging parenting strategies known to reduce risk for adolescent alcohol use.

Drawing on geocoded and survey data from the parents of Black, Latinx, and White youth who participated in Follow‐up 2 of the Adolescent Brain Cognitive Development (ABCD) Study, we aimed to identify and characterize subgroups of parents of middle‐school‐aged youth with respect to perceptions and behaviors related to youth alcohol access. Toward that goal, we derived subgroups based on parent‐reported data on whether any adults or youth in the household use alcohol, establishment of rules and penalties for violations of rules around their child's alcohol use, storage of alcohol in the home, and perception of their child's ease of access to alcohol. We then assessed for distinctions across subgroups by race/ethnicity, socioeconomic status (SES) indicators, community type (urban, suburban, or rural), and level of neighborhood advantage. We hypothesized that, consistent with findings from Ennett et al. (2016), three or more profiles would emerge. That is, drawing on an even wider range of indicators of alcohol specific parenting in a larger sample that includes Latinx youth as well as Black and White youth, we expected to find evidence of patterns beyond a dichotomy of overall restrictive (high restrictions paired with low access) and overall permissive (low restrictions paired with high access). We further hypothesized that, given the greater prevalence of abstention from alcohol among Black compared to Latinx and White adults (SAMHSA, 2022), Black youth would be overrepresented in profiles characterized by low access, more specifically, the absence of drinkers in the household.

## METHODS

### Sample and procedures

#### Analytic sample

The analytic sample was composed of youth who participated in Follow‐up 2 of the ABCD Study (see ABCD Study Overview and Procedures). Given our focus on Black, Latinx, and White youth, only youth identified as members of one of those racial/ethnic groups were included in analyses. Race/ethnicity was categorized according to the ABCD Study's method, using a combination of parent‐reported youth race and youth ethnicity (Latinx/Hispanic or Non‐Hispanic/Latinx). All individuals endorsing Latinx/Hispanic ethnicity were categorized as Latinx. Thus, those categorized as “White” identified as Non‐Latinx ethnicity and as White race; those categorized as “Black” identified as Non‐Latinx ethnicity and as Black/African American race. Gender also followed the ABCD categorization, using parent‐reported child's gender identity (youth did not self‐report gender identity at this wave) as “boy,” “girl,” or “other” gender. The composition of the analytic sample (*n* = 9586) with respect to gender was 47.50% girl, 51.32% boy, and 0.32% other gender. With respect to race/ethnicity, it was 14.29% Black, 25.97% Latinx, and 59.74% White. The mean age was 12.05 (SE = 0.00 [rounding to two decimal points]).

#### ABCD study overview and procedures

The ABCD Study is an ongoing multisite longitudinal study of adolescent health and cognitive development [https://abcdstudy.org]. Study design and ascertainment are described in detail in prior publications (Garavan et al., 2018). Briefly, from 2016 to 2018, 21 assessment sites in the United States recruited youth aged 9 or 10 and the child's primary caregiver (hereafter referred to as parent). Ascertainment targets were based on school enrollment data from the National Center for Education Statistics and data from the U.S. Census Bureau's annual American Community Survey. Probability sampling was applied to target schools within catchment areas. Protocols were approved by a centralized Institutional Review Board. Assent from the child as well as written informed consent from a parent were obtained at the time of enrollment.

#### Assessments

Comprehensive surveys were administered to the youth and parent in person (other than in 2020 and 2021, when they were administered online to accommodate pandemic conditions). Surveys covered demographic information, including socioeconomic status and race/ethnicity, and multiple health related domains: substance use, mental health, physical conditions, family history of major health problems, and a range of cultural and environmental constructs. Neurocognitive performance tasks were conducted as well but will not be described, as the present investigation did not draw on those data. The current study drew on survey data from Follow‐up 2 and geocoded data from baseline. The ABCD Study created geocoded variables based on location of youth's primary residence at baseline and included them in the publicly available data set.

### Measures

#### Alcohol restrictions and access

ABCD measures of restrictions and access were derived from the literature on parental protective strategies against youth alcohol use (Arthur et al., 2007; Dishion et al., 2003).

##### Parents' establishment of rules about alcohol use

Parents were asked, “Which statement best describes the rules for your child's drinking in your house?” Given the skewness toward very strict rules for youth, as expected at this young age, responses were coded dichotomously (yes or no). The response “I haven't made rules yet about my child drinking” was coded as no. The other five responses, representing varying levels of strictness (including the decision not to set any restrictions on their child's drinking), were all coded yes. Among those coded yes, the proportion endorsing each option was as follows: “My child is not allowed to drink under any circumstances”: 96.9%, “My child is allowed to drink at home but only on special occasions and under parent supervision”: 2.33%, “My child can have a drink when he/she asks for one”: 0.17%, “My child can drink at home whenever he/she wants to”: 0.12%, and “I don't set rules about my child's drinking”: 0.46%.

##### Penalties for violating rules about alcohol use

Parents who reported setting rules about their child's alcohol use were asked the follow‐up question, “Do you have penalties for violating family rules about drinking?” with “Yes” and “No” as possible responses. The variable was coded dichotomously (yes/no) according to responses from parents who set rules and as no for those who were skipped out because they reported that they had not yet set rules (and therefore, by definition, had not established penalties for rule violations).

##### Drinkers in the household

Parents were asked whether any adults or youth in the household consume alcohol, using separate questions. The two items were coded dichotomously (yes/no).

##### Storage of alcohol in the home

Parents who reported that either adults or youth in the household consume alcohol were asked how alcohol is stored in the home. Parents who responded “No” to both questions about drinkers in the household were skipped out of the alcohol storage questions. The 5‐level alcohol storage ordinal variable was coded from least to most secure (1 = least to 5 = most secure), based on the following four response options: “Visible and unlocked (countertop tabletop fridge easy to reach drawer),” “Not visible and unlocked (cabinet hard to reach drawer hidden),” “Locked cabinet drawer or storage area,” “Outside the home,” with participants who reported no drinkers in the household assigned a value of 5. The assignment of a value reflecting the lowest degree of household alcohol access was consistent with the embedded assumption by ABCD investigators (who created the skip logic patterns within the survey) that households without drinkers do not store alcohol.

##### Parent's perceived ease of youth access to alcohol

Parents' perceptions of their child's access to alcohol was assessed by asking, “If your child wanted to get some beer, wine, or hard liquor, how easy would it be for them to get some?” The variable was treated as ordinal (1–4), with higher values reflecting lower access, based on the response options, “Very easy,” “Sort of easy,” “Sort of hard,” and “Very hard.”

#### Covariates

##### Socioeconomic status (SES)

SES was assessed via household income and primary parent's educational attainment, represented in models as 3‐level and 5‐level variables, respectively (see Table 2), consistent with prior ABCD studies (e.g., Bagot et al., 2022).

##### Community type and neighborhood disadvantage

Geocoded data based on parent‐reported residential history, specifically, youth's primary residence at the time of baseline assessment, were linked to census‐tract data to code community type as urban, suburban/town, or rural and calculate the area deprivation index (ADI). The ADI is a standard indicator of neighborhood disadvantage that is derived from multiple neighborhood‐level statistics on such factors as educational attainment, income, employment, household utilities (e.g., telephone, plumbing), and home values. The values on the ADI reflect national‐level percentiles (1 to 100), with higher values reflecting greater deprivation. In the present investigation, we followed the common practice of analyzing ADI quartiles, that is, ≤25th, 26th–50th, 51st–75th, and ≥76th percentiles.

### Analysis plan

Latent profile analysis (LPA) was applied to the data, using the adjusted three‐step modeling procedure (Bakk & Vermunt, 2016) in Latent GOLD version 6.0 (Vermunt & Magidson, 2005, 2013) to identify distinct profiles representing parental restrictions and youth access to alcohol, along with profile correlates. The LPA model included six parent‐reported indicators: rules about alcohol use, penalties for breaking rules, adult alcohol use in the household, youth alcohol use in the household, parental perception of youth access to alcohol, and storage of alcohol. Using full information maximum likelihood estimation to accommodate missing data (Vermunt & Magidson, 2005), the LPA models tested the fit of 1 to 8 latent profiles, specifying 200 random starts and 1000 iterations. The LPA models accounted for ABCD's complex sampling approach. Analyses accounted for nesting of cases within families and sites and included ABCD's propensity‐based population sampling weight (Heeringa & Berglund, 2020) to account for sample ascertainment.

As the first step in the 3‐step procedure, the best‐fitting model was selected by considering multiple criteria, including the Bayesian Information Criterion [BIC; lower values indicate better fit; (Henson et al., 2007)], distinctiveness of the profiles (Collins & Lanza, 2010), and minimizing profiles with low prevalence (e.g., <10%) because low prevalence profiles may be less replicable (Nylund‐Gibson & Choi, 2018). The best‐fitting model provided estimates of parameters such as probabilities of latent profile membership and item response probabilities for each profile. Differences between the profiles on each of the six indicators were examined using Wald tests, with posthoc pairwise comparisons between profiles used to identify specific differences between profiles on the indicators.

After selecting the best‐fitting model, step two involved saving the probabilities of profile assignment to account for classification error when examining profile correlates (step three). Profile correlates were youth race/ethnicity, youth gender, youth age, parental education level, household income, community type (urban, suburban, or rural), and ADI quartile. In step 3, maximum likelihood estimation was used to examine profile correlates (Bakk & Vermunt, 2016), with standard errors estimated using nonparametric bootstrapping (Bakk et al., 2016; Lanza et al., 2013; Vermunt & Magidson, 2021). Wald tests, with posthoc pairwise comparisons between profiles, were used to identify specific differences between profiles on the correlates (Vermunt & Magidson, 2021). To account for multiple testing (inclusive of pairwise comparisons across correlates), we applied the Benjamini‐Hochberg procedure, adopting a 5% false discovery rate (Benjamini & Hochberg, 1995).

## RESULTS

### Profile identification

**FIGURE 1:**
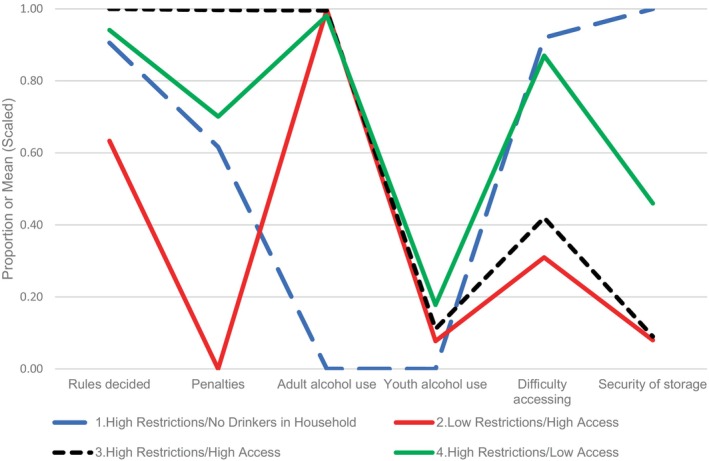
Indicators of restrictions and access by profile.

Results of LPA model fit testing 1 through 8 profiles are shown in Supplemental Table A and Figure 1. The 4‐profile solution was selected based on meeting a higher number of model fit criteria than other models tested. Specifically, although the 8‐profile solution had the lowest BIC, models with five or more profiles included profiles with low prevalence (<5% of cases), which suggested that the models may have low replicability. The model with four profiles was selected over the 3‐profile model based on lower BIC, distinctiveness of the classes, and adequate distribution of cases across classes. Values for the proportion of parents reporting that rules about alcohol use have been established, there are penalties for violating rules, some adults in the household drink alcohol, and some youth in the household drink alcohol, along with means for perceived youth access and security of alcohol storage (with higher values indicating lower access), are reported by profile in Table 1. Profile 1 (High Restrictions/No Drinkers in Household, 32.18%) was distinguished by the absence of drinkers in the household. Profile 2 (Low Restrictions/High Access, 29.58%) was characterized by the lowest endorsement of having rules about alcohol use, the absence of penalties for violating rules, the highest perceived ease of youth obtaining alcohol, and a lack of secure storage in the household. Profile 3 (High Restrictions/High Access, 26.38%) was distinguished by universal endorsement of having rules as well as penalties for violating rules but high perceived ease of youth obtaining alcohol and a lack of secure storage in the household. Profile 4 (High Restrictions/Low Access, 11.86%) was distinguished by the highest endorsement of youth in the household drinking alcohol and the most secure storage.

**TABLE 1:** Latent profile indicators.

	Total Sample *N* = 9586	1. High restrictions/No drinkers in household (32.18%)	2. Low restrictions/High access (29.58%)	3. High restrictions/High access (26.38%)	4. High restrictions/Low access (11.86%)	Wald statistic	*p‐*value	Significant paired comparisons
Rules decided: %	85.43	90.57	63.35	100.00	94.11	19,358.16	<0.001	1–2, 1–3, 2–3, 2–4, 3–4
Penalties: %	54.46	61.65	0.01	99.69	70.09	16,901.78	<0.001	1–2, 1–3, 1–4, 2–3, 2–4, 3–4
Adult alcohol use %	67.40	0.01	99.73	99.55	98.10	5050.94	<0.001	1–2, 1–3, 1–4, 2–4, 3–4
Youth alcohol use: %	7.32	0.00	7.73	11.11	17.79	11,954.82	<0.001	1–2, 1–3, 1–4, 2–3, 2–4, 3–4
Access: Mean (SE)	2.80 (0.02)	3.75 (0.02)	1.92 (0.03)	2.26 (0.04)	3.62 (0.05)	1141.41	<0.001	1–2, 1–3, 1–4, 2–3, 2–4, 3–4
Storage: Mean (SE)	2.69 (0.02)	5.00 (0.00)	1.31 (0.01)	1.36 (0.02)	2.82 (0.07)	30,325.06	<0.001	1–2, 1–3, 1–4, 2–4, 3–4

*Note*: Access coded: 1 = very easy, 2 = sort of easy, 3 = sort of hard, 4 = very hard; Storage coded: 1 = visible, unlocked; 2 = not visible and unlocked; 3 = locked cabinet or storage area; 4 = outside the home; 5 = no alcohol in the household (no one drinks). Significant paired comparisons = Paired profile differences statistically significant at *p* < 0.05 post false discovery rate adjustment.

Abbreviation: SE, standard error.

Wald tests, generated in the LPA, revealed statistically significant differences across profiles for all six indicators. Posthoc pairwise comparisons were conducted to identify specific profile differences. With the exceptions of Profiles 2 (Low Restrictions/High Access) and 3 (High Restrictions/High Access) on adult alcohol use and alcohol storage and Profiles 1 (High Restrictions/No Drinkers in Household) and 4 (High Restrictions/Low Access) on whether rules had been decided, all paired comparisons were statistically significant after false discovery rate adjustment. Results of paired comparisons are reported in Supplemental Table B and shown in the final column in Table 1.

### Profile correlates

**FIGURE 2:**
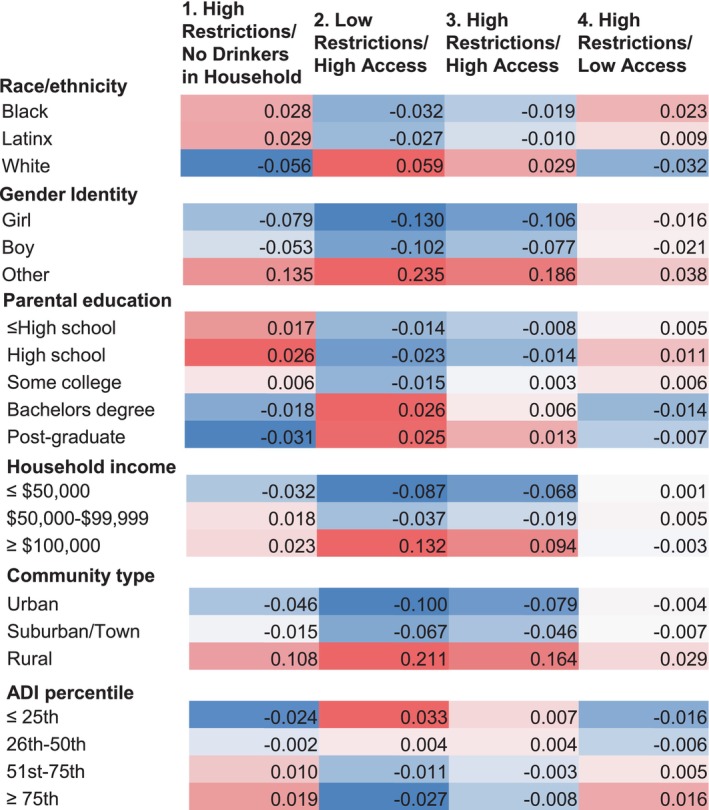
Demographic and neighborhood correlates of profiles. Differences between observed and expected percentages: Blue cells indicate overrepresented and red cells indicate underrepresented compared with the model expectation. Deeper hues indicate greater degree of deviation.

The distributions of race/ethnicity, gender, parental education, household income, community type, and ADI quartile, along with mean youth age, are shown by profile in Table 2. Wald tests, generated in the LPA, revealed statistically significant differences after false discovery rate adjustment across profiles for all seven correlates (Table 2). Results of posthoc paired comparisons are reported in Supplemental Table C. All pairwise comparisons of the profiles were significant for race/ethnicity. The magnitude of associations is depicted in the form of heat maps in Figure 2. The heat maps show differences between observed and expected proportions, where red cells indicate overrepresentation of cases relative to expected values and blue cells indicate underrepresentation of cases relative to expected values. Darker hues in the heat maps reflect a greater deviation from the expected value.

**TABLE 2:** Latent profile correlates.

	Total sample, *N* = 9586	1. High restrictions/No drinkers in household (32.18%)	2. Low restrictions/High access (29.58%)	3. High restrictions/High access (26.38%)	4. High restrictions/Low access (11.86%)	Wald statistic	*p‐*value	Significant paired comparisons
Race/ethnicity (%)						178.39	<0.001	1–2, 1–3, 1–4, 2–3, 2–4, 3–4
Black	14.29	22.86	3.45	7.15	33.95			
Latinx	25.97	34.90	16.78	22.09	33.30			
White	59.74	42.24	79.77	70.76	32.75			
Gender identity (%)						11.94	0.027	2–4, 3–4
Girl	47.50	49.04	46.50	46.82	47.32			
Boy	51.32	49.96	52.29	51.53	52.12			
Other	0.32	0.14	0.40	0.58	0.02			
Age: Mean (SE)	12.05 (0.00^a^)	12.04 (0.01)	12.01 (0.01)	12.10 (0.02)	12.05 (0.03)	14.85	0.002	1–2, 2–3, 2–4
Parental education (%)						97.92	<0.001	1–2, 1–3, 2–3, 2–4, 3–4
<High school	5.64	10.94	0.96	2.69	9.49			
High school	13.64	21.76	5.97	8.26	22.75			
Some college	14.85	16.76	9.84	15.84	19.92			
Bachelor's degree	25.70	20.14	34.54	27.83	13.98			
Post‐graduate	39.56	29.78	48.13	44.65	33.40			
Household income (%)						270.34	<0.001	1–2, 1–3, 2–3, 2–4, 3–4
<$50,000	32.80	52.66	13.10	21.33	53.60			
$50,000–$99,999	29.35	27.22	31.60	28.86	30.58			
≥$100,000	34.98	16.31	53.26	47.30	12.60			
Community type (%)						53.71	<0.001	1–2, 1–3, 2–3, 3–4
Urban	37.10	44.93	33.70	28.19	44.16			
Suburban/Town	39.56	35.55	38.80	46.32	37.24			
Rural	8.53	6.80	8.54	11.00	7.69			
ADI Percentile (%)						34.89	<0.001	1–2, 2–3, 2–4
≤25th	25.73	18.25	36.91	28.27	12.49			
26th–50th	32.86	32.17	34.27	34.54	27.46			
51st–75th	18.55	21.57	14.69	17.38	22.65			
>76th	15.95	21.80	6.96	12.79	29.51			

*Note*: Percentages within a profile may not sum to 100 due to missing data. Percentage missing values for household income, community type, and ADI variables differed by racial/ethnic group, but missingness was not consistently higher across variables for any given racial/ethnic group. Significant paired comparisons = Paired profile differences statistically significant at *p <* 0.05 post false discovery rate adjustment.

Abbreviation: SE, standard error.

^a^ Rounded to two decimal points.

As shown in Table 2 and depicted in the heat maps, parents of Black and Latinx youth were overrepresented in the High Restrictions/No Drinkers in Household (1) and High Restrictions/Low Access (4) profiles, whereas parents of White youth were overrepresented in the Low Restrictions/High Access (2) and High Restrictions/High Access (3) profiles. For both parental education and household income (the two family‐level SES indicators), all pairwise comparisons of the profiles were significant except for High Restrictions/No Drinkers in Household (1) versus High Restrictions/Low Access (4). In those two profiles, the majority of parents had a household income under $50,000 and nearly one‐third had a high school or lower level of education. Regarding community type, the High Restrictions/High Access (3) profile differed from all the others, with the lowest proportion of families in this profile living in urban areas and the highest in rural areas. The Low Restrictions/High Access (2) profile differed from all others on the ADI, with greater than one‐third of families in the most advantaged and under 7% in the least advantaged quartile.

## DISCUSSION

The current study took a novel approach to understanding parents' restrictions and perceptions of their child's access to alcohol in Black, Latinx, and White families, deriving distinct profiles and characterizing them with respect to sociodemographic and neighborhood factors. Four profiles (i.e., subgroups) emerged, supporting the notion that parents' perspectives and behaviors cannot be simply divided into restrictive and permissive. As hypothesized, the subgroups were further distinguished by degree of restrictions on youth alcohol use and perceived accessibility: high restrictions paired alternatively with low access and high access, low restrictions paired with high access, and a fourth profile (1: High Restrictions/No Drinkers in Household) in which parents reported no alcohol use in the household. Profile comparisons on race/ethnicity, SES indicators, community type, and neighborhood advantage highlighted subgroup distinctions that could inform tailoring of parent‐level prevention efforts.

In addition to the defining distinctions, commonalities across subgroups were observed. Consistent with prior studies of parental rule‐setting regarding alcohol use (Janssen et al., 2014), across subgroups, the majority of parents (85.43%) had decided on rules for youth alcohol use, and, as would be expected among families with preadolescent‐aged children, few parents reported that any youth in the household drank alcohol. Notably, the subgroup with the highest endorsement of alcohol use by some youth in the home (4: High Restrictions/Low Access: 17.79%) also reported the most secure storage of alcohol in the home, potentially reflecting action in this small minority of parents to curb youth alcohol use that had come to their attention.

### Defining features of subgroups

The establishment of penalties for breaking rules about alcohol use, perception of their child's ease of obtaining alcohol, and storage of alcohol in the home varied across subgroups to a greater degree than rule‐setting. Seemingly counterintuitively, the second lowest endorsement of establishing penalties—after the Low Restrictions/High Access (2) subgroup, in which penalties were universally absent—was the High Restrictions/No Drinkers in Household (1) subgroup. This finding highlights the importance from a prevention perspective of understanding the context in which parents establish restrictions and consequences of violating them. In families where no household members drink, choosing not to set rules and/or penalties for violating rules may not be indicative of a generally permissive attitude toward youth alcohol use. Rather, it may be due to parents not feeling the need to establish rules or set penalties, given the absence of alcohol in the home.

Parents' perceived ease of their child's access to alcohol was the indicator with the greatest range in responses, spanning from easy (2: Low Restrictions/High Access) to very hard (1: High Restrictions/No Drinkers in Household and 4: High Restrictions/Low Access). The variability in perceived access, and importantly, its pairing with the likelihood of taking measures to restrict access, suggests the importance of considering parents' perceptions. The majority of parents in the two subgroups who indicated it would be easy for their child to obtain alcohol also reported that the alcohol they keep in their home is visible and unlocked. The interpretability of this finding is limited by the absence of information on the parents' concerns that their children—who were about 12 years old at the time of assessment—may actually want to obtain alcohol. Parents who do not believe that their child is interested in using alcohol may simply be reporting that it would be easy for their child to obtain alcohol since they do not securely store it, as such precautions are not necessary. Other parents may believe that if their child is interested in using alcohol, they could obtain it outside the home, so there is limited value in locking it up. This possibility is consistent with prior work documenting increased permissive attitudes among parents who became aware of their child's drinking (Glatz et al., 2012), although, notably, that work was conducted with parents of older youth, so it may not apply to the current sample.

### Sociodemographic characteristics of subgroups

Each of the four subgroups differed significantly from each other with respect to racial/ethnic composition, and, except for the two subgroups defined by low access (1: High Restrictions/No Drinkers in Household and 4: High Restrictions/Low Access), they also differed on parental education and household income. Consistent with the higher prevalence of alcohol abstention among Black and Latinx compared to White adults (SAMHSA, 2022) and among lower SES families (Kerr et al., 2016), parents of Black and Latinx youth and parents with relatively low educational attainment and income were overrepresented in the High Restrictions/No Drinkers in Household (1) subgroup. They were also overrepresented in the other subgroup defined by low access (4: High Restrictions/Low Access), suggesting that Black and Latinx youth and youth from modest socioeconomic backgrounds are especially likely to live in households where alcohol is relatively difficult to access, based on both parent perception and reported storage in the home. This was a somewhat unexpected finding for Latinx youth, given prior reports of greater access to alcohol among Latinx youth relative to youth from other racial/ethnic groups (Lardier Jr. et al., 2018). However, inconsistencies may be attributable to the differing developmental periods captured in the prior research and the current study: mid to late adolescence versus preadolescence (age 12), when access is generally quite limited. By contrast, the two high access subgroups had a disproportionate number of White youth and youth from socioeconomically advantaged backgrounds, consistent with higher prevalence of alcohol use among White relative to Black and Latinx adults (SAMHSA, 2022) and in high versus lower SES populations (Kerr et al., 2016).

Some subgroup distinctions were observed with respect to community type and neighborhood advantage as well. For example, the High Restrictions/High Access (3) profile differed significantly from the other three, with the highest proportion of suburban and rural dwelling families. The least restrictive subgroup, the Low Restrictions/High Access (2) subgroup, was distinguished from the other three as residing in the most advantaged neighborhoods. This latter finding seems to contradict the well‐documented high density of alcohol outlets in disadvantaged neighborhoods (Morrison et al., 2016; Tobler et al., 2009) but needs to be considered in a developmental context. In the middle school years, alcohol access is likely better captured by the immediate environment, e.g., the presence of drinkers and storage of alcohol in the home, than proximity to alcohol retailers.

### Limitations

Certain limitations should be kept in mind when interpreting study results. First, parental perspectives and behaviors related to youth alcohol access change as their children age, so results cannot be generalized beyond the preadolescent years. Second, our analytic sample drew data exclusively from parents of Black, Latinx, and White youth; findings may not generalize to members of other racial/ethnic groups. Third, although we used national rather than sample‐specific ADI percentiles to capture participants' neighborhood‐level advantage and applied weighting variables to account for ascertainment to reduce potential bias, socioeconomically advantaged families were overrepresented and rural families were underrepresented in the sample. Fourth, the skip logic pattern integrated into the ABCD Study survey for participants reporting that no household members drink alcohol makes the assumption that these families do not store alcohol to serve nonhousehold members, an assumption that may not be accurate for some families.

### Future directions

Study findings suggest a number of potential future directions, several of which we anticipate pursuing with the ABCD cohort, including parallel examinations of parental restrictions and perceptions of their child's access to combustible cigarettes, e‐cigarettes, and cannabis. As the cohort ages and data from later developmental stages become available, we also anticipate exploring transitions in these alcohol‐specific parenting behaviors across youth developmental periods. Importantly, we will examine their links to youth alcohol use, which we did not pursue in the present study, given that alcohol use was very limited, low level (i.e., sips), and nearly universally parent‐sanctioned. This next stage of research, linking profiles of alcohol‐specific parenting to youth alcohol outcomes, has the potential to inform parent‐level targeted prevention efforts that capitalize on community norms to support and enhance implementation of protective strategies.

## FUNDING INFORMATION

This research was supported by grants R01MD016922 from the National Institute on Minority Health and Health Disparities (NIMHD) and R00AA025394 and P60AA006282 from the National Institute on Alcohol Abuse and Alcoholism (NIAAA). The content is solely the responsibility of the authors and does not necessarily represent the official views of the National Institutes of Health.

## CONFLICT OF INTEREST STATEMENT

The authors have no conflict of interest to declare.

## Supporting information


Appendix S1.

## Data Availability

The data that support the findings of this study are openly available in NIMH Data Archive at https://nda.nih.gov/study.html?id=2313.
