# Supplementary material for: Parents' perspectives and behaviors regarding their child's access to alcohol: Variation by race/ethnicity, socioeconomic status, and neighborhood
Source: Alcohol Clin Exp Res (Hoboken). 2024 Dec 19;49(1):234–43. doi: 10.1111/acer.15498 (PMC11740169; doi:10.1111/acer.15498)
Supplement: Supplementary file 1 — Appendix S1. [file ACER-49-234-s001.docx]

Supplemental Table A. Comparison of model fit, testing 1-8 profile solutions

| **Profiles** | **LL** | **BIC(LL)** | **AIC(LL)** | **N par** | **L²** | **df** | **p-value** | **Class. Err.** | **Entropy R²** |
| --- | --- | --- | --- | --- | --- | --- | --- | --- | --- |
| 1 | -41401.2 | 82903.16 | 82824.31 | 11 | 18791.22 | 629 | <0.001 | 0.00 | 1.00 |
| 2 | -34319.8 | 68804.57 | 68675.55 | 18 | 4628.45 | 622 | <0.001 | 0.02 | 0.93 |
| 3 | -33212.8 | 66654.87 | 66475.67 | 25 | 2414.58 | 615 | <0.001 | 0.07 | 0.86 |
| 4 | -32891.4 | 66076.23 | 65846.85 | 32 | 1771.76 | 608 | <0.001 | 0.08 | 0.88 |
| 5 | -32577.3 | 65512.16 | 65232.61 | 39 | 1143.51 | 601 | <0.001 | 0.08 | 0.87 |
| 6 | -32456.8 | 65335.31 | 65005.58 | 46 | 902.49 | 594 | <0.001 | 0.09 | 0.85 |
| 7 | -32395.7 | 65277.27 | 64897.37 | 53 | 780.27 | 587 | <0.001 | 0.10 | 0.84 |
| 8 | -32346.2 | **65242.57** | 64812.49 | 60 | 681.40 | 580 | 0.002 | 0.20 | 0.73 |

Notes: Bolded value=lowest BIC. LL=Log Likelihood, BIC=Bayesian Information Criterion, AIC=Akaike Information

Criterion, N Par= Number of Parameters, L2= Log Likelihood2, df=degrees of freedom, Class Err= Classification errors

Supplemental Table B. Paired comparisons between profiles on the 6

indicators that define the profiles

| **Indicator** | **Paired profile comparison** | **Wald (df)** | ***p*-value** |
| --- | --- | --- | --- |
| Rules decided | 1 vs. 2 * | 307.51 (1) | <0.001 |
|  | 1 vs. 3 * | 7062.95 (1) | <0.001 |
|  | 1 vs. 4 | 3.98 (1) | 0.046 |
|  | 2 vs. 3 * | 18613.13 (1) | <0.001 |
|  | 2 vs. 4 * | 79.85 (1) | <0.001 |
|  | 3 vs. 4 * | 1023.95 (1) | <0.001 |
| Penalties | 1 vs. 2 * | 16027.88 (1) | <0.001 |
|  | 1 vs. 3 * | 110.28 (1) | <0.001 |
|  | 1 vs. 4 * | 8.29 (1) | 0.004 |
|  | 2 vs. 3 * | 835.08 (1) | <0.001 |
|  | 2 vs. 4 * | 5019.25 (1) | <0.001 |
|  | 3 vs. 4 * | 89.24 (1) | <0.001 |
| Adult alcohol use | 1 vs. 2 * | 833.57 (1) | <0.001 |
|  | 1 vs. 3 * | 1118.80 (1) | <0.001 |
|  | 1 vs. 4 * | 2008.10 (1) | <0.001 |
|  | 2 vs. 3 | 0.58 | 0.440 |
|  | 2 vs. 4 * | 8.93 (1) | 0.003 |
|  | 3 vs. 4 * | 6.13 (1) | 0.013 |
| Youth alcohol use | 1 vs. 2 * | 6445.44 (1) | <0.001 |
|  | 1 vs. 3 * | 7607.24 (1) | <0.001 |
|  | 1 vs. 4 * | 4949.14 (1) | <0.001 |
|  | 2 vs. 3 * | 10.27 (1) | 0.001 |
|  | 2 vs. 4 * | 33.53 (1) | <0.001 |
|  | 3 vs. 4 * | 10.86 (1) | <0.001 |
| Access | 1 vs. 2 * | 977.88 (1) | <0.001 |
|  | 1 vs. 3 * | 656.99 (1) | <0.001 |
|  | 1 vs. 4 * | 9.08 (1) | 0.003 |
|  | 2 vs. 3 * | 50.66 (1) | <0.001 |
|  | 2 vs. 4 * | 283.89 (1) | <0.001 |
|  | 3 vs. 4 * | 219.49 (1) | <0.001 |
| Storage | 1 vs. 2 * | 774.54 (1) | <0.001 |
|  | 1 vs. 3 * | 737.26 (1) | <0.001 |
|  | 1 vs. 4 * | 30117.49 (1) | <0.001 |
|  | 2 vs. 3 | 3.88 (1) | 0.049 |
|  | 2 vs. 4 * | 41.59 (1) | <0.001 |
|  | 3 vs. 4 * | 36.73 (1) | <0.001 |

Notes: Profile 1=Abstainer; Profile 2=Low Restrictions/High Access;

Profile 3=High Restrictions/High Access; Profile 4=High Restrictions/

Low Access. * Statistically significant at *p<*0.05 after false discovery

rate adjustment

Supplemental Table C. Paired comparisons between latent profile correlates

| **Correlates** | **Paired profile comparison** | **Wald (df)** | ***p*-value** |
| --- | --- | --- | --- |
| Race/ethnicity | 1 vs. 2 * | 136.21 (2) | <0.001 |
|  | 1 vs. 2 * | 65.67 (2) | <0.001 |
|  | 1 vs. 4 * | 14.90 (2) | 0.001 |
|  | 2 vs. 3 * | 15.85 (2) | 0.001 |
|  | 2 vs. 4 * | 123.38 (2) | <0.001 |
|  | 3 vs. 4 * | 73.26 (2) | <0.001 |
| Gender identity | 1 vs. 2 | 4.89(2) | 0.087 |
|  | 1 vs. 3 | 6.23 (2) | 0.044 |
|  | 1 vs. 4 | 4.42 (2) | 0.110 |
|  | 2 vs. 3 | 0.79 (2) | 0.670 |
|  | 2 vs. 4 * | 9.04 (2) | 0.011 |
|  | 3 vs. 4 * | 9.94 (2) | 0.007 |
| Age | 1 vs. 2 * | 10.06 (1) | 0.002 |
|  | 1 vs 3 | 0.68 (1) | 0.410 |
|  | 1 vs. 4 | 0.19 (1) | 0.660 |
|  | 2 vs. 3 * | 18.81 (1) | <0.001 |
|  | 2 vs. 4 * | 6.03 (1) | 0.014 |
|  | 3 vs. 4 | 0.03 (1) | 0.860 |
| Parental education | 1 vs. 2 * | 69.87 (4) | <0.001 |
|  | 1 vs. 3 * | 35.77 (4) | <0.001 |
|  | 1 vs. 4 | 8.50 (4) | 0.075 |
|  | 2 vs. 3 * | 22.40 (4) | <0.001 |
|  | 2 vs. 4 * | 35.55 (4) | <0.001 |
|  | 3 vs. 4 * | 10.63 (4) | 0.031 |
| Household income | 1 vs. 2 * | 181.01 (2) | <0.001 |
|  | 1 vs. 3 * | 157.09 (2) | <0.001 |
|  | 1 vs. 4 | 4.55 (2) | 0.100 |
|  | 2 vs. 3 * | 7.37 (2) | 0.025 |
|  | 2 vs. 4 * | 53.37 (2) | <0.001 |
|  | 3 vs. 4 * | 42.90 (2) | <0.001 |
| Community type | 1 vs. 2 * | 11.09 (2) | 0.004 |
|  | 1 vs. 3 * | 51.72 (2) | <0.001 |
|  | 1 vs. 4 | 0.91 (2) | 0.630 |
|  | 2 vs. 3 * | 20.76 (2) | <0.001 |
|  | 2 vs. 4 | 1.50 (2) | <0.001 |
|  | 3 vs. 4 * | 13.90 (2) | <0.001 |

| Supplemental Table C. Paired comparisons between latent profile correlates (continued) | | | |
| --- | --- | --- | --- |
| **Correlates** | **Paired profile comparison** | **Wald (df)** | ***p*-value** |
| Area Deprivation Index | 1 vs. 2 * | 13.01 (3) | 0.005 |
|  | 1 vs. 3 | 4.77 (3) | 0.190 |
|  | 1 vs. 4 | 8.16 (3) | 0.043 |
|  | 2 vs. 3 * | 18.65 (3) | <0.001 |
|  | 2 vs. 4 * | 18.63 (3) | <0.001 |
|  | 3 vs. 4 | 3.58 (3) | 0.310 |

Notes: Profile 1= High Restrictions/No Drinkers in Household;

Profile 2=Low Restrictions/High Access; Profile 3=High Restrictions/High

Access; Profile 4=High Restrictions/Low Access. * Statistically significant

at *p<*0.05 after false discovery rate adjustment
